# Association Between Individual and Intimate Partner Factors and Cervical Cancer Screening in Kenya

**DOI:** 10.5888/pcd15.180182

**Published:** 2018-12-13

**Authors:** Tapati Dutta, Laura Haderxhanaj, Jon Agley, Wasantha Jayawardene, Beth Meyerson

**Affiliations:** 1 Department of Applied Health Science, Indiana University School of Public Health, Bloomington, Indiana; 2 Indiana Prevention Resource Center, Institute for Research on Addictive Behavior, Department of Applied Health Science, Indiana University School of Public Health, Bloomington, Indiana; 3 Rural Center for AIDS/STD Prevention, Department of Applied Health Science, Indiana University School of Public Health, Bloomington, Indiana

## Abstract

**Introduction:**

Cervical cancer is the most prevalent cancer among women in Kenya. Although cervical cancer screening could reduce illness and death, screening rates remain low. Kenyan women’s individual characteristics and intimate partner factors may be associated with cervical cancer screening; however, a lack of nationally representative data has precluded study until recently. The objective of our study was to examine individual and intimate partner factors associated with cervical cancer screening in Kenya.

**Methods:**

We conducted secondary data analysis of responses by women who completed the cervical cancer screening and domestic violence questions in the Kenya Demographic and Health Survey, 2014 (N = 3,222). By using multivariable regression analyses, we calculated the association of cervical cancer screening with age, religion, education, wealth, recent exposure to family planning on television, head of household’s sex, and experience of intimate partner violence.

**Results:**

Rates of cervical cancer screening among women in Kenya increased with age. The wealthiest women and women with post-secondary education had greater odds of reporting being screened for cervical cancer than the poorest women and uneducated women. Christians and women exposed to prevention messaging on television had higher odds of screening than Muslims and women with no exposure. Victims of intimate partner violence had lower odds of being screened than women who had not experienced intimate partner violence.

**Conclusion:**

Identified barriers to screening in this sample mirror previous findings, though with additional nuances. Model fit data and theoretical review suggest that additional, unmeasured variables may contribute to variability in cervical cancer screening rates. Inclusion of additional variables specific to cervical cancer in future national surveys could strengthen the ability to identify factors associated with screening.

## Introduction

Cervical cancer is the most prevalent cancer among women in Kenya (1). The World Health Organization (WHO) calls for early detection and treatment of precancerous lesions to prevent cervical cancer and reduce disease-related illness and death (2). However, the effectiveness of cervical cancer screening in reducing population-level cervical cancer rates depends on access and uptake, quality of screening, adequacy of follow-up, and diagnosis and treatment (3). WHO notes that low- and middle-income countries struggle to implement early detection programs (2), often because of obstacles such as poverty, lack of information and knowledge, and health care infrastructure (4).

Available options for cervical cancer screening in Kenya are Papanicolaou testing and visual inspection of the cervix with acetic acid followed by visual inspection with Lugol's iodine. The Loop Electrosurgical Excision Procedure for removal of abnormal or cancerous cervical cells is available at some national and district hospitals (5). Kenyan national guidelines recommend screening women aged 25 to 49 and women younger than 25 who are at high risk for cervical cancer (6). However, despite the availability of screening options and national efforts to increase screening, use of cervical cancer screening services remains low in Kenya, with the lowest rates among rural women and those belonging to nomadic livestock-herding tribes (7–9). Concurrently, Kenya has experienced an increase in cervical cancer cases, from 2,454 in 2012 to 4,802 (crude incidence rate = 22.4) in 2016. An estimated 4,100 Kenyan women are expected to develop cervical cancer, and an estimated 3,300 will die from the disease by 2025 if prevention efforts are not increased (1).

Studies undertaken in sub-Saharan Africa showed that cervical cancer screening uptake is a complex issue associated with multiple individual and interpersonal factors (10). Individual factors include age, education level, access and affordability (including transportation to cervical cancer screening facilities), attitude toward personal health, and fear of a cervical cancer diagnosis (11,12). Previous studies also suggested a positive association between women’s autonomy and cervical cancer screening, but the relationship is indirect. Women’s autonomy is associated with individual factors such as education, income, control over household finances, knowledge of the signs and symptoms of cervical cancer, and intimate partner agency, such as communication between partners and freedom from threat of intimate partner violence (2,13). Some studies in sub-Saharan Africa also examined religious affiliation as it relates to women’s autonomy and health care decision making and access (14). In these countries, a complex interplay of individual and intimate partner factors, along with organizational factors such as infrastructure and technical, human, and health care resources, are necessary to sustain effective cervical cancer screening programs (15).

Concomitant with studies in sub-Saharan Africa, studies exploring factors associated with the uptake of cervical cancer screening in Kenya showed that women’s perceived susceptibility to cervical cancer increased their tendency to be screened for the disease (10,12). However, these studies were mostly conducted in selected health care facilities or in counties and did not use a national data set. To supplement this knowledge base, this study used a nationally representative Kenyan data set to examine the association between cervical cancer screening and selected individual and intimate partner factors identified by previous research that have both theoretical and research-driven linkages to women’s autonomy and to cervical cancer screening uptake.

## Methods

### Sampling

Data were from the Kenya Demographic Health Survey (KDHS), 2014, which was administered from May 2014 through October 2014 by the Kenya National Bureau of Statistics (KNBS) in partnership with numerous national and international agencies and foundations. This was the sixth and most recent demographic health survey conducted in Kenya since 1989. It was the first to provide representative data for all 47 Kenyan counties and national and regional findings and was also the first to include specific questions about cervical cancer screening (16). The sample for the 2014 KDHS consisted of 5,360 clusters split into 4 equal subsamples and was drawn by using stratified probability proportional to size sampling from a master sampling frame developed by KNBS. Data for our study were drawn from the full women’s survey, which was administered to women aged 15 to 49 (N = 14,741). Of those women, 70.1% (n = 10,333) had ever heard of cervical cancer and responded to the question about whether they had been screened for cervical cancer. Separately, 38.5% (n = 5,672) of the full women’s sample were selected to take the domestic violence module; 15 women were excluded a priori because they either could not be interviewed for privacy or other unspecified reasons. Because the study’s purpose was to examine the association between cervical cancer screening and individual and intimate partner variables, our sample included women who had been in a union (ie, reported being married or living with a partner or widowed, divorced, or separated), responded to the cervical cancer screening questions (ie, were part of the 10,333-woman subsample), and completed the domestic violence module (ie, were part of the 5,672-woman subsample) for a final sample of 3,222 women. Additional details about the survey are available (16) (Figure).

**Figure:**
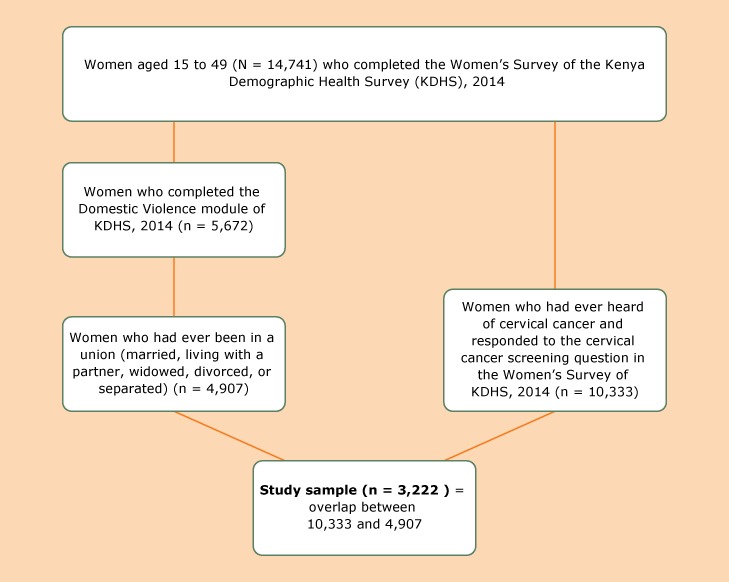
Study sample of women selected from the Kenya Demographic Health Survey, 2014, to analyze the association between cervical cancer screening and women’s individual characteristics and intimate partner factors.

Some women selected to complete the domestic violence module who were currently or had previously been in a union were ineligible for the study sample because they either 1) had not heard of cervical cancer or 2) were not sure whether they had heard of it and thus, because of the survey structure, could not indicate whether they had received screening. To investigate how women having no knowledge of cervical cancer might affect sample selection, researchers ran a preliminary logistic regression with having heard of cervical cancer as the outcome variable (1 = no/unsure, 2 = yes). The Hosmer–Lemeshow test was used to test for goodness of fit for the logistic regression models. All preparatory analyses indicated appropriate model fit and nonviolation of statistical assumptions.

### Measures

The outcome variable, screening for cervical cancer, was binary (1 = reported ever having been screened, 0 = reported never having been screened). Some independent variables were selected on the basis of our literature review, including age, religion, education, wealth quintile, and sex of the head of household. Others were chosen because of similarity with theoretical covariates that were not captured in KDHS (for example, exposure to family planning on television is within the same conceptual domain as general knowledge about cervical cancer, though less precise; intimate partner violence is a component of interpartner agency). However, some variables identified in previous research were not measured in the recent KDHS (7), including attitude toward personal health and fear of cervical cancer, though potentially related to cervical cancer screening.

Age was measured as a continuous variable within the allowable range for participants (15–49). Religion was measured as a categorical variable with 4 possible choices (0 = Roman Catholic, 1 = Protestant/other Christian, 2 = Muslim, 3 = no religion), and education was measured as a categorical variable with 6 possible choices (0 = no education, 1 = incomplete primary, 2 = complete primary, 3 = incomplete secondary, 4 = complete secondary, 5 = higher). In Kenya, primary education encompasses the first 8 years, and secondary is the next 4 years (equivalent to US high school). Wealth index was captured as a categorical variable with 5 quintiles calculated by KDHS (0 = poorest, 1 = poor, 2 = middle, 3 = richer, 4 = richest). Sex of the head of household was a binary variable (1 = male, 2 = female), as was having heard of family planning on television in the last few months (0 = no, 1 = yes).

Being subjected to intimate partner violence was constructed as a dummy variable (0 = no intimate partner violence, 1 = any intimate partner violence) on the basis of responses to questions modified from the Conflict Tactics Scale (CTS): “Did your husband/partner ever do any of the following things to you,” with 10 item choices, such as “slap you and punch you with his fist or something that could hurt you.” Information about question wording and location within the survey instrument is available in the KDHS survey monograph (16).

### Statistical analysis

All statistical analyses were completed with SPSS version 24 (IBM Corp). All categorical independent variables (religion, education, wealth, exposure to family planning on television, sex of head of household, and intimate partner violence) were analyzed by using Wald χ^2 ^tests of independence to determine associations with cervical cancer screening for inclusion in the regression model. A model was used to assess the association, expressed as adjusted odds ratios (AORs), between the dependent variable — cervical cancer screening — and the 6 independent variables (age, religion, education, wealth, exposure to family planning on television, and intimate partner violence). All but one bivariate test was significant (critical α = 0.05, Bonferroni’s adjusted α = 0.008). Thus, all the selected variables except sex of head of household (*P* = .98) were included in the multivariable logistic regression model to compute AORs. Age, the only continuous independent variable, was tested in a single-variable logistic regression model to calculate an unadjusted odds ratio. Age was significant, so it was also included in the final model.

The independent variables were tested for multicollinearity before analysis, and no problematic patterns emerged (all tolerance values were >0.20). The single noncategorical variable, age, was normally distributed, with no univariate outliers and no skewness or kurtosis values exceeding an absolute value of 1.

## Results 

Our first analysis, designed to identify characteristics of women included in the sample, was not the primary purpose of the study. In brief, we found that the odds of having heard of cervical cancer (and thus being eligible for study inclusion) increased substantially with each new level of educational attainment relative to no education, whereas all other variables and levels, excepting Roman Catholic religion and intimate partner violence, had modest but significant association with knowledge of cervical cancer. In the sample of 3,222 women who were eligible for our study, 18.2% reported having received cervical cancer screening (Table 1). The mean age of women who reported being screened was 33.8, compared with 31.8 for those who reported not being screened. Among those screened, nearly all women (97.3%) self-identified as some variant of Christianity, whereas those who reported not having been screened were more religiously heterogeneous (89.9% Christian). Among those screened, most women (52.8%) had at least some primary school education, about a third belonged to the richest wealth quintile (30.5%), and more than half (54.3%) were exposed to family planning messaging on television. In addition, 31.9% of women who reported being screened for cervical cancer reported intimate partner violence, whereas 38.6% of women who reported never having been screened reported intimate partner violence.

**Table 1:** Screening for Cervical Cancer Among Kenyan Women Aged 15 to 49 Currently or Previously in a Marriage or Domestic Partnership (N = 3,222), Kenya Demographic Health Survey, 2014

Characteristic	Screened for Cervical Cancer, n (%)	Not Screened for Cervical Cancer, n (%)	χ^2a^	*P* Value
**Total sample^b^**	587 (18.2)	2,635 (81.8)	—	—
**Individual Factors**				
**Age, y, mean (standard deviation)**	33.81 (7.9)	31.76 (7.8)	—	—
**Religion**				
Protestant or other Christian	423 (72.1)	1,834 (69.6)	38.15	<.001
Roman Catholic	148 (25.2)	535 (20.3)		
Muslim	9 (1.5)	221 (8.4)		
No religion	7 (1.2)	44 (1.7)		
**Education**				
No education	16 (2.7)	195 (7.4)	66.86	<.001
Incomplete primary	134 (22.8)	767 (29.1)		
Complete primary	176 (30.0)	795 (30.2)		
Incomplete secondary	76 (12.9)	314 (11.9)		
Complete secondary	90 (15.3)	373 (14.2)		
Higher	95 (16.2)	191 (7.2)		
**Sex of household head**				
Male	386 (65.8)	1,734 (65.8)	0.00	.98
Female	201 (34.2)	901 (34.2)		
**Wealth index^c^**				
Poorest	49 (8.3)	478 (18.1)	94.40	<.001
Poor	101 (17.2)	618 (23.5)		
Middle	102 (17.4)	545 (20.7)		
Richer	156 (26.6)	558 (21.2)		
Richest	179 (30.5)	436 (16.5)		
**Heard of family planning on television in the last few months**				
Yes	319 (54.3)	998 (37.9)	53.8	<.001
No	268 (45.7)	1,636 (62.1)		
**Intimate Partner**				
**Experienced intimate partner violence by husband/partner**				
Yes	187 (31.9)	1,017 (38.6)	9.32	.002
No	400 (68.1)	1,618 (61.4)		

Abbreviation: —, not applicable.

a Results of χ^2^ test.

b Values are number (percentage) unless otherwise indicated.

c The wealth index in the Kenya Demographic Health Survey is constructed by using household asset data collected in the survey’s Household Questionnaire.

The model used in our study to assess the association, expressed as AORs, between the dependent variable – cervical cancer screening – and the 6 independent variables (age, religion, education, wealth, exposure to family planning on television, and intimate partner violence) was a significant, modest improvement over the constant-only model (χ^2 ^= 193.34, *P* < .001; −2LL improved from 3,058.91 to 2,864.77). The Hosmer and Lemeshow Test was nonsignificant, indicating a likelihood of good model fit.

Each additional year of age increased the odds of being screened for cervical cancer by 1.04 relative to the previous year of age (*P* < .001) (Table 2). Muslim women had 5 times lower odds than Protestants or other Christians of having been screened (*P* < .001). Women who reported an education level higher than secondary had 1.93 times greater odds than women who reported no education of having been screened for cervical cancer (*P* = .04). In addition, women who were in the top 2 wealth quintiles (richer and richest) had significantly greater odds of having been screened for cervical cancer than women in the lowest wealth quintile (poorest), 1.95 times (*P* = .001) for richer, and 2.53 times (*P* < .001) for richest. Women who reported having heard family planning messaging on television within the past few months had 1.34 times greater odds of having been screened than those who had not (*P* = .01). Finally, women who experienced at least one type of intimate partner violence had 1.28 times lower odds (the reciprocal of AOR .78) of having been screened for cervical cancer than women who had not experienced intimate partner violence (*P* < .001).

**Table 2:** Multivariable Logistic Regression Analysis of Women’s Individual and Intimate Partner Measures and Cervical Cancer Screening Among Kenyan Women Aged 15 to 49 (N = 3,222), Kenya Demographic Health Survey, 2014

Variable	Adjusted Odds Ratio (95% Confidence Interval)	β	*P* Value
**Individual**			
**Age, y^a^**	1.04 (1.03–1.05)	0.04	<.001
**Religion^a^**			
Protestant or other Christian	Reference		
Roman Catholic	1.22 (0.98–1.51)	0.20	.08
Muslim^a^	0.20 (0.10–0.40)	−1.62	<.001
No religion	0.98 (0.43–2.24)	−0.25	.95
**Education**			
No education	Reference		
Incomplete primary	1.42 (0.80–2.51)	0.35	.23
Complete primary	1.40 (0.79–2.48)	0.33	.26
Incomplete secondary	1.40 (0.76–2.58)	0.33	.29
Complete secondary	1.17 (0.64–2.16)	0.16	.61
Higher education^a^	1.93 (1.03–3.64)	0.66	.04
**Wealth index**			
Poorest	Reference		
Poor	1.32 (0.90–1.92)	0.27	.15
Middle	1.42 (0.97–2.08)	0.35	.07
Richer^a^	1.95 (1.34–2.86)	0.67	.001
Richest^a^	2.53 (1.67–3.84)	0.93	<.001
**Heard of family planning on television, last few months^a^^,^ b**	1.34 (1.07–1.68)	0.29	.01
**Intimate Partner**			
Intimate partner violence^a^	0.78 (0.64–0.95)	−0.25	<.001

a Indicates significance at *P* < .05.

b Reference answer was no.

## Discussion

Ours is one of the few studies in Africa and the first in Kenya to use national data to examine the association between women’s individual characteristics and those of their intimate partner relationships and cervical cancer screening. Although our study showed that a low percentage of Kenyan women, 18%, were being screened for cervical cancer, screening rates appear to be increasing. A 2003 study found that only 3.5% of Kenyan women had been screened for cervical cancer (8). Nevertheless, the increase in screening rates remained much lower than the national target of 75% by the year 2009 (4).

Our findings mirror other studies in developing countries reporting that older age is associated with increased odds of being screened for cervical cancer (2,17). This could be a function of increased sensitization through prevention messaging over a period of time. However, lower uptake of cervical screening among young women is a matter of concern because screening has the greatest impact when initiated early (3).

Our study found a strong relationship between religious affiliation and cervical cancer screening, mirroring work from Nigeria that reported that use of antenatal services was more likely among Christian women than among their Muslim counterparts (18). However, the relationship between an individual’s religion, religiosity, and sexual and contraception behaviors is nuanced and merits further investigation (14,15). Studies of cervical cancer screening among Muslim women demonstrated that screening practices were perceived as incompatible with cultural and religious values (19), such as cancer being a function of God’s will (20). These studies suggest that religious coping may function as an extension of prevailing gender constructs in these countries, which are patriarchal, and where talking about female genitalia and sexuality is taboo (21).

The association between formal education and cervical cancer screening was not significant among women with no education until reaching a threshold of an education level higher than secondary education, at which point education was associated with higher odds of reported screening. This finding is conceptually similar to results from a study conducted in India (22). The directionality in this area has not been universal, however. One study in India suggested that women’s autonomy and prevention decision making were not related to education level (23). The association between wealth quintile and cervical cancer screening is likewise unsurprising; prior research in Mexico showed wealth quintile to be one of the most consistent determinants of cervical cancer screening (24). In our study, the association was strong, but only significant for the top 2 wealth quintiles relative to the lowest. Given acute poverty in the arid and semi-arid parts of Kenya and prevailing patriarchal norms, women have inequitable access to economic assets (16). Thus, even when screening is provided free of cost by the Kenyan Government, out-of-pocket expenses to reach the facility for screening and associated loss of wages may present barriers to access even for those of moderate means (25). Research in Ethiopia examined a similar relationship in the context of patriarchal norms and found that women were reluctant to ask for payment for cervical cancer screening expenses from their husbands or partners (26).

Although our study suggested a positive association between exposure to family planning messaging on television and cervical cancer screening, the mechanism of this relationship cannot be determined given the available data; viewing family planning messages on television has multiple possible moderating and mediating effects, such as access to television (independent of the type of messaging) (27) and the quality of the family planning content. To provide more data, a question specific to receipt of cervical cancer information could be included in future iterations of KDHS.

Finally, in our study, experiencing intimate partner violence was associated, to a moderate degree, with decreased odds of having been screened for cervical cancer. However, because ours was a cross-sectional study, directionality could not be assumed. This, too, is consistent with previous research that demonstrated that control imposed by an abusive partner and the associated limited access to financial support can restrict women’s ability to seek cervical cancer services (28). The same study also found that seeking cervical cancer screening is often perceived by the husband or partner as a consequence of adultery, resulting in intimate partner violence. In our study, however, the association between intimate partner violence and screening was moderate relative to the magnitude of other associations, especially religious affiliation. Given the sensitive nature of this topic, the degree to which survey participants accurately reported their experiences of intimate partner violence is uncertain. Intimate partner violence is socially stigmatized, and women may be concerned that reporting intimate partner violence might increase the risk of additional violence from an intimate partner. The relationship between these variables is also unlikely to be unidimensional and may include other psychosocial factors such as stress, social support, self-esteem (29), and diverse variations in concepts of family and of women’s autonomy.

Our study had limitations. First, as a cross sectional study, it was not possible to assess causality. In addition, the overall multivariable logistic regression model met tests of model goodness-of-fit and improved on the constant-only model by a modest amount. Although these variables were important to understanding cervical cancer screening among the study population, they did not represent the totality of variance within that behavior. The model did not control for multiple residual confounding variables, because data were not available from KDHS. A survey more directly targeting cervical cancer may facilitate more robust model fit. Also, as noted in Methods, the ability to respond to the question about cervical cancer screening was not randomly distributed among women. Finally, using only intimate partner violence indicators for intimate partner relations might not have captured the complex gender relations that influenced cervical cancer screening behaviors and might have provided biased estimates of the affect that autonomy has on preventive health care (30).

The low rate of cervical cancer screening in Kenya represents a significant preventable health burden for women. The results of our study suggest that, despite low cervical cancer screening rates, Kenyan women’s barriers to screening mirror those found in other national and local studies, with some additional nuances (eg, the lack of association between moderate income increases and screening). These findings reinforce specific issues that might be addressed to advance cervical cancer screening (eg, the impact of post-secondary education on screening) as well as some broader conceptual issues that might facilitate health care improvement, such as facilitating responses to sensitive intimate partner–related questions. We recommend including additional cervical cancer–specific questions in future national surveys.
